# Publisher Correction: Structural basis of LRPPRC–SLIRP-dependent translation by the mitoribosome

**DOI:** 10.1038/s41594-024-01402-7

**Published:** 2024-09-19

**Authors:** Vivek Singh, J. Conor Moran, Yuzuru Itoh, Iliana C. Soto, Flavia Fontanesi, Mary Couvillion, Martijn A. Huynen, L. Stirling Churchman, Antoni Barrientos, Alexey Amunts

**Affiliations:** 1 Science for Life Laboratory, Department of Biochemistry and Biophysics, Stockholm University, Solna, Sweden; 2 Medical Scientist Training Program, Department of Biochemistry and Molecular Biology, University of Miami Miller School of Medicine, Miami, FL USA; 3 Blavatnik Institute, Department of Genetics, Harvard Medical School, Boston, MA USA; 4 Department of Medical BioSciences, Radboud University Medical Center, Nijmegen, The Netherlands; 5 Department of Neurology, University of Miami Miller School of Medicine, Miami, FL USA; 6 Present Address: Department of Biological Sciences, Graduate School of Science, University of Tokyo, Tokyo, Japan; 7 Present Address: Westlake University, Hangzhou, China

**Keywords:** Cryoelectron microscopy, RNA

Correction to: *Nature Structural & Molecular Biology* 10.1038/s41594-024-01365-9, published online 12 August 2024.

In the version of the article initially published, the text in the abstract now reading “cytochrome c oxidase subunit 1 and 2” originally read “cyclooxygenase 1 and 2”. In the first paragraph of the “LRPPRC is recruited for translation of mRNAs” section, the text now reading “cytochrome c oxidase subunit 1 (COX1)” originally read “cyclooxygenase 1”. These corrections have now been made to the HTML and PDF versions of the article.

