# Supplementary material for: Structural basis of LRPPRC–SLIRP-dependent translation by the mitoribosome
Source: Nat Struct Mol Biol. 2024 Aug 12;31(12):1838–47. doi: 10.1038/s41594-024-01365-9 (PMC11637978; doi:10.1038/s41594-024-01365-9)
Supplement: Supplementary file 1 — Reporting Summary [file 41594_2024_1365_MOESM1_ESM.pdf]

nature portfolio

Corresponding author(s): ALEXEY AMUNTS

Last updated by author(s): Apr 1, 2024

Reporting Summary

Nature Portfolio wishes to improve the reproducibility of the work that we publish. This form provides structure for consistency and transparency in reporting. For further information on Nature Portfolio policies, see our Editorial Policies and the Editorial Policy Checklist.

Statistics

For all statistical analyses, confirm that the following items are present in the figure legend, table legend, main text, or Methods section.

n/a	Confirmed	
☐	☒	The exact sample size (n) for each experimental group/condition, given as a discrete number and unit of measurement
☐	☒	A statement on whether measurements were taken from distinct samples or whether the same sample was measured repeatedly
☒	☐	The statistical test(s) used AND whether they are one- or two-sided<br>*Only common tests should be described solely by name; describe more complex techniques in the Methods section.*
☒	☐	A description of all covariates tested
☒	☐	A description of any assumptions or corrections, such as tests of normality and adjustment for multiple comparisons
☐	☒	A full description of the statistical parameters including central tendency (e.g. means) or other basic estimates (e.g. regression coefficient) AND variation (e.g. standard deviation) or associated estimates of uncertainty (e.g. confidence intervals)
☒	☐	For null hypothesis testing, the test statistic (e.g. F, t, r) with confidence intervals, effect sizes, degrees of freedom and P value noted<br>*Give P values as exact values whenever suitable.*
☒	☐	For Bayesian analysis, information on the choice of priors and Markov chain Monte Carlo settings
☒	☐	For hierarchical and complex designs, identification of the appropriate level for tests and full reporting of outcomes
☒	☐	Estimates of effect sizes (e.g. Cohen's d, Pearson's r), indicating how they were calculated

Our web collection on statistics for biologists contains articles on many of the points above.

Software and code

Policy information about availability of computer code

Data collection	The datasets were collected EPU 1.9 software on FEI Titan Krios (FEI/Thermofischer) transmission electron microscope operated at 300 keV with a slit width of 20 eV on a GIF quantum energy filter (Gatan). A K2 Summit detector (Gatan) was used at a pixel size of 0.83 Å (magnification of 165,000x) with a dose of 29-32 electrons/Å2 fractionated over 20 frames. A defocus range of -0.6 to -2.8 µm was used.
Data analysis	Movie frames were aligned and averaged by global and local motion corrections by the program RELION-3.0.2. Contrast transfer function (CTF) parameters were estimated by gctf. Particles were picked, 2D and 3D classified by RELION 3.0.2. Particle separation into optics groups was done in RELION-3.1. Beam-tilt, magnification anisotropy and higher-order (trefoil and fourth-order) aberrations were corrected in RELION-3.1. The models were manually built with Coot 0.9 and stereochemical refinement was performed using phenix.real_space_refine in the PHENIX 1.18 suite. Optical densities of the immunoreactive bands were measured using the Histogram function of the Adobe Photoshop 22.0.0 software in digitalized images.

For manuscripts utilizing custom algorithms or software that are central to the research but not yet described in published literature, software must be made available to editors and reviewers. We strongly encourage code deposition in a community repository (e.g. GitHub). See the Nature Portfolio guidelines for submitting code & software for further information.

Data

Policy information about availability of data

All manuscripts must include a data availability statement. This statement should provide the following information, where applicable:

- Accession codes, unique identifiers, or web links for publicly available datasets
- A description of any restrictions on data availability
- For clinical datasets or third party data, please ensure that the statement adheres to our policy

The atomic coordinates were deposited in the RCSB Protein Data Bank, and EM maps have been deposited in the Electron Microscopy Data bank under accession numbers 8ANY and EMD-15544. The atomic coordinates that were used in this study: 6ZTJ (E.coli 70S-RNAP expressome complex in NusG); 6ZTN (E.coli 70S-RNAP expressome complex in NusG); 1RKJ (human Nucleolin); 5WWE (human hnRNPA2/B1); 1CVJ (Poly-adenylate binding protein, PABP). For building LRPPRC-SLIRP module, the initial model of the full length LRPPRC was obtained from AlphaFold2 Protein Structure Database (Uniprot ID P42704). The initial model of SLIRP was obtained from Alphafold2 Protein Structure Database (Uniprot ID Q9GZT3).

Human research participants

Policy information about studies involving human research participants and Sex and Gender in Research.

Reporting on sex and gender	N/A
Population characteristics	N/A
Recruitment	N/A
Ethics oversight	N/A

Note that full information on the approval of the study protocol must also be provided in the manuscript.

Field-specific reporting

Please select the one below that is the best fit for your research. If you are not sure, read the appropriate sections before making your selection.

☒ Life sciences ☐ Behavioural & social sciences ☐ Ecological, evolutionary & environmental sciences

For a reference copy of the document with all sections, see nature.com/documents/nr-reporting-summary-flat.pdf

Life sciences study design

All studies must disclose on these points even when the disclosure is negative.

Sample size	A total of 994,919 particles were pooled and analyzed. No statistical analyses has been performed. The number of cryo-EM particles in the single dataset collected was the number of particles available. No predetermined sample size was used for other experiments.
Data exclusions	For cryo-EM structure determination, particles that were not mitochondrial ribosome were discarded by classification, since they cannot contribute to reconstruction.
Replication	Cryo-EM structures were successfully obtained from three preliminary datasets.
Randomization	Cryo-EM map resolution estimates by Fourier Shell Correclation were performed using half-maps from random half-sets.
Blinding	Raw micrographs or particle images are not categorical data. Particles are randomly assigned into half-sets for image processing; hence no blinding is applicable.

Reporting for specific materials, systems and methods

We require information from authors about some types of materials, experimental systems and methods used in many studies. Here, indicate whether each material, system or method listed is relevant to your study. If you are not sure if a list item applies to your research, read the appropriate section before selecting a response.

Materials & experimental systems

n/a	Involved in the study
☐	☒ Antibodies
☐	☒ Eukaryotic cell lines
☒	☐ Palaeontology and archaeology
☒	☐ Animals and other organisms
☒	☐ Clinical data
☒	☐ Dual use research of concern

Methods

n/a	Involved in the study
☒	☐ ChIP-seq
☒	☐ Flow cytometry
☒	☐ MRI-based neuroimaging

Antibodies

Antibodies used

Primary antibodies against β-ACTIN (dilution 1:2,000; Proteintech; Rosemont, IL; 60008-1-Ig), ATP5A (1:1000; Abcam; Cambridge, MA; ab14748), CORE2 (1:1,000; Abcam; Cambridge, MA; ab14745), COX1 (dilution 1:2,000; Abcam; Cambridge, MA; ab14705), LRPPRC (dilution 1:1,000; Proteintech; Rosemont, IL; 21175-1-AP), NDUFA9 (1:1000; Proteintech; Rosemont, IL; 20312-1-AP), SDHA (1:1,000; Proteintech; Rosemont, IL; 14865-1-AP) or SLIRP (1:1000; Abcam; Cambridge, MA; ab51523). Horseradish peroxidase-conjugated anti-mouse or anti-rabbit IgGs were used as secondary antibodies (dilution 1:10,000; Rockland; Limerick, PA).

Validation

Describe the validation of each primary antibody for the species and application, noting any validation statements on the manufacturer's website, relevant citations, antibody profiles in online databases, or data provided in the manuscript.

Eukaryotic cell lines

Policy information about cell lines and Sex and Gender in Research

Cell line source(s)

HEK293S-derived cells line T501 was originally purchased from Thermofisher Scientific.

Authentication

No authentication is required as these cells are Zeocin and Blasticidin resistant.

Mycoplasma contamination

Cell line tested negative for mycoplasma contamination.

Commonly misidentified lines
(See ICLAC register)

No commonly misidentified cell lines were used in the study.
