# Supplementary figures and images for: Structural basis of LRPPRC–SLIRP-dependent translation by the mitoribosome
Source: Nat Struct Mol Biol. 2024 Aug 12;31(12):1838–47. doi: 10.1038/s41594-024-01365-9 (PMC11637978; doi:10.1038/s41594-024-01365-9)

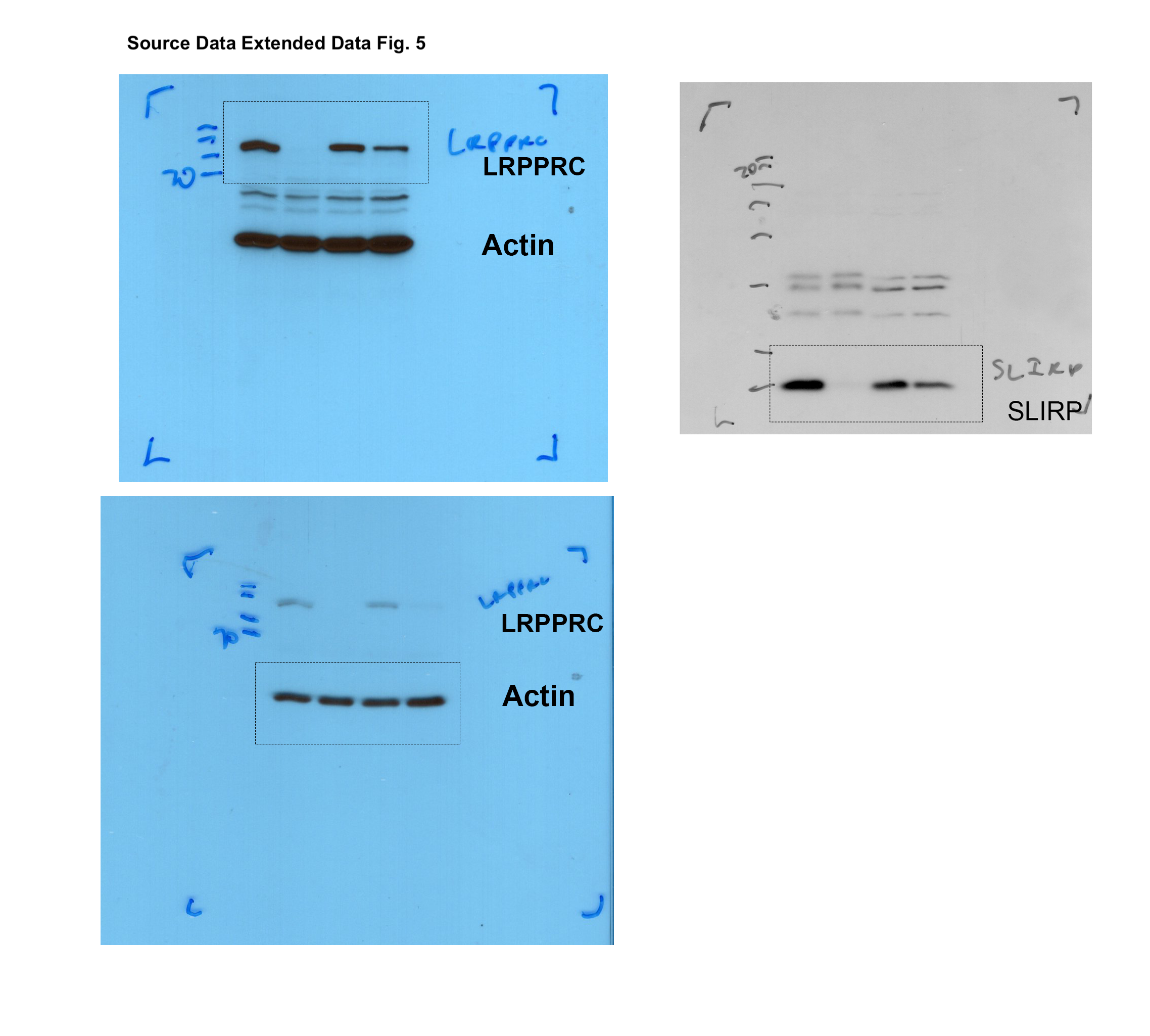

Supplement: Supplementary file 5 — Unprocessed western blots. [file 41594_2024_1365_MOESM5_ESM.tiff]

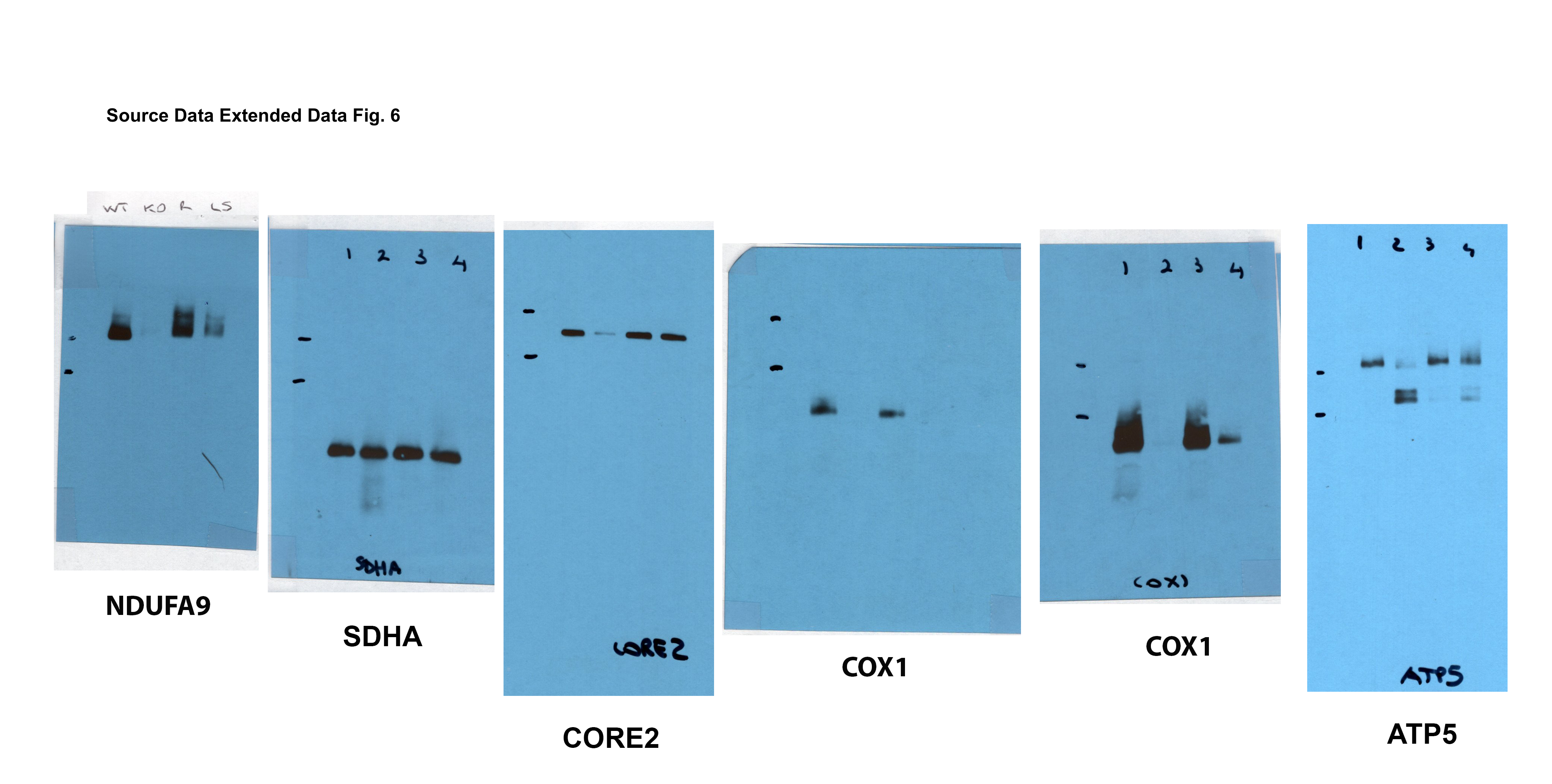

Supplement: Supplementary file 6 — Unprocessed western blots. [file 41594_2024_1365_MOESM6_ESM.tiff]
